# Identification of long noncoding RNA RP11-89K21.1 and RP11-357H14.17 as prognostic signature of endometrial carcinoma via integrated bioinformatics analysis

**DOI:** 10.1186/s12935-020-01359-9

**Published:** 2020-06-24

**Authors:** Lingling Gao, Xin Nie, Wenchao Zhang, Rui Gou, Yuexin Hu, Yue Qi, Xiao Li, Qing Liu, Juanjuan Liu, Bei Lin

**Affiliations:** 1 Department of Obstetrics and Gynaecology, Shengjing Hospital of China Medical University, No.36 Sanhao Street, Heping District, Shenyang, 110004 Liaoning China; 2 Key Laboratory of Maternal–Fetal Medicine of Liaoning Province, Key Laboratory of Obstetrics and Gynecology of Higher Education of Liaoning Province, Liaoning, China

**Keywords:** Endometrial carcinoma, Long noncoding RNA, Prognosis, Competing endogenous RNA, Bioinformatics analysis

## Abstract

**Background:**

Endometrial carcinoma (EC) is one of the most common malignant tumors in gynecology. The potential functions and mechanisms of long noncoding RNAs (lncRNAs) in the occurrence and progression of EC remains unclear. It’s meaningful to explore lncRNAs signature for providing prognostic value of EC.

**Methods:**

The differentially expressed lncRNAs and their prognostic values in EC were investigated based on The Cancer Genome Atlas (TCGA) database; the transcriptional factors (TFs), the competing endogenous RNA (ceRNA) mechanism, functional regulatory network and immune infiltration of RP11-89K21.1 and RP11-357H14.17 were further explored by various bioinformatics tools and databases.

**Results:**

We firstly identified high expression of RP11-89K21.1 and RP11-357H14.17 were closely associated with shorten overall survival (OS) and poor prognosis in patients with EC. We also elucidated the networks of transcription factor and co-expression genes associated with RP11-89K21.1 and RP11-357H14.17. Furthermore, the ceRNA network mechanism was successfully constructed through 2 lncRNAs (RP11-89K21.1 and RP11-357H14.17), 11 miRNAs and 183 mRNAs. Functional enrichment analysis revealed that the targeting genes of RP11-89K21.1 and RP11-357H14.17 were strongly associated with microRNAs in cancer, vessel development, growth regulation, growth factor and cell differentiation, and involved in pathways including pathways in cancer, microRNAs in cancer and apoptotic signaling pathway.

**Conclusions:**

We demonstrated for the first time that RP11-89K21.1 and RP11-357H14.17 may play crucial roles in the occurrence, development and malignant biological behavior of EC, and can be regarded as potential prognostic biomarkers for EC.

## Background

Endometrial carcinoma (EC) is one of the most common types of malignancies in the female reproductive system, accounting for 20% to 30% of the total number of malignant tumors in female genital tract. In some developed countries, the incidence of EC is higher than that of cervical cancer, which ranks first among gynecological tumors [1]. In recent years, the incidence of EC in China has increased year by year and ranked second of gynecological cancer, showing younger trend and severely threatening the physical and mental health of women [2]. At present, the treatment of endometrial cancer included surgery, radiotherapy, and chemotherapy, while patients with advanced EC may have distant metastasis, postoperative recurrence, and poor prognosis. Therefore, exploring potential biomarkers closely associated with the occurrence and development of EC are of great value for early diagnosis and targeted therapy of endometrial carcinoma.

Long noncoding RNAs (lncRNAs) are a group of non-coding RNAs with more than 200 bp in length with no or limited protein-coding function, which were first discovered in mice in 2002 and lack of specific and complete open reading frame [3]. LncRNAs, as important regulators of transcription and translation, have been found not only involved in physiological and pathological processes, including chromatin remodeling, transcription, post-transcriptional translation, cell proliferation, differentiation and metabolic reprogramming [4, 5], but also playing a pivotal role in the occurrence and development of malignant tumors [6]. Abnormal expression of lncRNAs can affect the development and progression of many kinds of malignant tumors, such as prostate cancer [7], ovarian cancer [8], breast cancer [9] and gastric cancer [10]. In recent years, a variety of lncRNAs have been identified to be essential for the initiation, progression and malignant behaviors of endometrial carcinoma [11]. High expression of MALAT1 [12], HOTAIR [13] and NEAT1 [14] were closely associated with the poor prognosis of EC and promoted the proliferation, metastasis and EMT of endometrial cancer cells. Other studies have shown that the expression of MEG3 [15] and FER1L4 [16] were decreased in EC, and high expression of MEG3 and FER1L4 inhibited the proliferation, migration and invasion of endometrial cancer cells. These studies suggest that lncRNAs play a crucial role in the prognosis and malignant biological behaviors of EC.

In this study, we investigated the differentially expressed lncRNAs in EC based on the Cancer Genome Atlas (TCGA) database and identified two lncRNA RP11-89K21.1 and RP11-357H14.17 and their correlation with the occurrence, development, prognostic value and functional regulatory network of EC. We also explored the upstream transcriptional regulatory factors, co-expression genes and binding proteins of lncRNAs and their relationship with immune infiltration. Furthermore, we explored their potential roles and molecular mechanisms in EC utilizing competing endogenous RNA (ceRNA) (lncRNA-miRNA-mRNA) hypothesis, which is extremely meaningful to provide a new strategy for early diagnosis and treatment of endometrial carcinoma.

## Materials and methods

### Screening for differentially expressed lncRNAs by circlncRNAnet

CirclncRNAnet (http://120.126.1.61/circlnc/index.php) [17] is an online tool for exploring lncRNA and circRNA chip or sequencing expression data integrated with more than 20 tumor types in TCGA database, which included several analysis modules, such as heat map, box diagram, co-expression scatter map, circos map, gene functional enrichment analysis, RBP-RNA binding protein network and miRNA network. We explored the differentially expressed lncRNAs in EC based on Uterine Corpus Endometrial Carcinoma (UCEC) with circlncRNAnet, and constructed Circos map and heat map of LncRNAs co-expression genes, Pearson’s correlation analysis was employed for exhibiting significant correlation between co-expressed genes and these lncRNAs (default: |r| > 0.5). A total of 10,978 lncRNAs were identified in the cohort and we obtained 121 dysregulated lncRNAs (77 upregulated and 44 downregulated).The screening criteria were defined as follows: |Log2 fold change | > 4 and *P* < 0.01.

### The prognosis of dysregulated lncRNAs analyzed with GEPIA and Kaplan–Meier plotter

GEPIA (http://gepia.cancer-pku.cn/) [18] is a newly web-based tool that contains sequencing expression data from 9736 tumor samples of 33 cancer types and 8587 normal samples. The database includes a variety of analysis modules such as differential gene expression analysis, survival and prognosis analysis, correlation analysis, as well as dimensionality reduction analysis. In this study, GEPIA database was employed to further analyze the expressionand prognostic value of differentially expressed lncRNAs in UCEC. The expression analysis of these genes performed by one-way ANOVA, and the filter criteria were as follows: |Log2FC| > 1, *P* value < 0.05, “median”, Hazards Ratio (HR) and 95% confidence interval. The Kaplan–Meier (KM) Plotter (http://kmplot.com) [19] is an effective tool for detecting the prognosis of patients with tumors. According to the expression of lncRNAs, patients with EC were divided into two groups: high and low expression group. The hazard ratio (HR) at a 95% confidence interval and log-rank *P*-values were also investigated online. The filter conditions were as follows: cancer: pan-cancer RNA-seq (Uterus corpus endometrial carcinoma); survival: overall survival (OS); follow-up threshold: 120 months.

### The cellular localization of lncRNAs

UCSC (https://genome-asia.ucsc.eduk/index.html) [20] provides a web-based interface that helps users browse gene information, view genome annotation assemblies and download gene sequences. LNCipedia (https://lncipedia.org) [21] is a freely available annotated database of human lncRNAs transcriptional sequences and structures, which utilizes secondary structure information to establish a standard and unified classification and naming system. The tool offers insights into functions of over 1500 human lncRNAs, including evaluating coding ability, predicting open reading frame and secondary structure. LncLocator (https://LncLocatorwww.csbio.sjtu.edu.cn/bioinf/lncLocator/) [22] is a free public platform to predict the subcellular localization of lncRNAs based on a stacked ensemble classifier. Only by utilizing lncRNA sequence information, the distribution proportion of lncRNA in 5 subcellular localizations, including cytoplasm, nucleus, ribosome, cytosol and exosome, can be quickly obtained. In this study, lncRNAs sequences information were detected by UCSC and LNCipedia databases, and the cellular localization of lncRNAs were then determined by LncLocator.

### Prediction and expression of candidate miRNA with AnnoLnc and starBase database

AnnoLnc (http://annolnc.cbi.pku.edu.cn) [23] is a web interface to systematically annotate newly identified human lncRNAs based on more than 700 data sources. The systematic annotation of lncRNAs cover a wide range of functions, including genome location, secondary structure, expression pattern, transcriptional regulation, miRNA interaction, protein interaction, genetic association and evolution. StarBase (http://starbase.sysu.edu.cn/) [24] provides an widely-used open-source platform for exploring ncRNA interactions based on 10,882 RNA sequence and 10,546 miRNA sequence of 32 cancer types, the platform can be used to perform the survival and differential expression analysis of miRNAs and lncRNAs. We predicted lncRNAs-binding miRNAs with AnnoLnc and further explored the expression of miRNAs in UCEC by starBase.

### Protein–protein interaction network and transcriptional regulatory network

GeneMANIA (http://www.genemania.org) [25] is a flexible and user-friendly platform that can predict gene function, analyze gene lists and sequence genes with function assays, which provides three main cases: single gene queries, multiple gene queries and network search. The online tool can be used to construct protein–protein interaction (PPI) network and protein-DNA interaction, investigate potential signal pathway, gene and protein expression and protein domains. We explored lncRNA-related proteins and transcriptional regulatory molecules with AnnoLnc, and visualized the functions and regulatory networks of these molecules using GeneMANIA.

### Construction of lncRNA–miRNA–mRNA regulatory network

miRTarBase (http://mirtarbase.mbc.nctu.edu.tw/php/index.php) [26] is a popular web interface which mainly collects miRNA target genes verified by different validation experiments and provides supportive evidences with literatures or assays. The database can be queried through different categories, such as miRNA, gene, disease, pathway and so on. Cytoscape [27] is a very powerful software for visualizing and analyzing network data, which allows users to construct many complex biological networks. Node and edge are the two core elements in the network diagram constructed by Cytoscape. miRNA target genes were explored by miRTarBase, and only those genes verified by at least one powerful experimental method were identified as miRNAs targets (reporter assay, Western blot or quantitative reverse transcription PCR). Cytoscape 3.7.1 was further employed to construct competing endogenous RNA (ceRNA) network (lncRNA-miRNA-mRNA).

### Functional enrichment analysis with Metascape

Metascape (http://metascape.org) [28] is a user-friendly and effective tool for comprehensively annotating and analyzing single or multiple genes lists, which integrates many authoritative database resources such as Gene Ontology (GO), Kyoto Encyclopedia of Genes and Genomes (KEGG), UniProt and Drugbank. We can not only complete pathway enrichment and biological process annotation, but also construct protein–protein interaction (PPI) networks with Metascape. In this study, Metascape was used to analyze the GO and KEGG enrichment of differentially expressed genes related to lncRNAs. Restrictions: *P* < 0.01, a minimum count of 3, enrichment factor > 1.5 were considered to be statistically significant. The PPI enrichment analysis in Metascape was based on the following databases: BioGrid, InWeb_IM and OmniPath. In addition, Molecular Complex Detection (MCODE) algorithm is applied to mine molecules with deeper network regulation relationships.

### Correlation between lncRNAs and immune infiltration analyzed with ImmLnc

ImmLnc (http://bio-bigdata.hrbmu.edu.cn/ImmLnc) [29] is an online analysis website for investigating the immune-related function of lncRNAs across 33 cancer types with high-throughput methods, Users can investigated the lncRNA-pathways, lncRNA-immune cell type’s correlation, and cancer-related lncRNAs. The ImmLnc serves as a valuable resource for exploring the lncRNA function and to further advance the identification of immunotherapy targets. In this study, we explored the correlation between lncRNAs and immune cell infiltration with ImmLnc.

## Results

### Identification of differentially expressed lncRNAs in Uterine Corpus Endometrial Carcinoma (UCEC) with circlncRNAnet and GEPIA

**Fig. 1:**
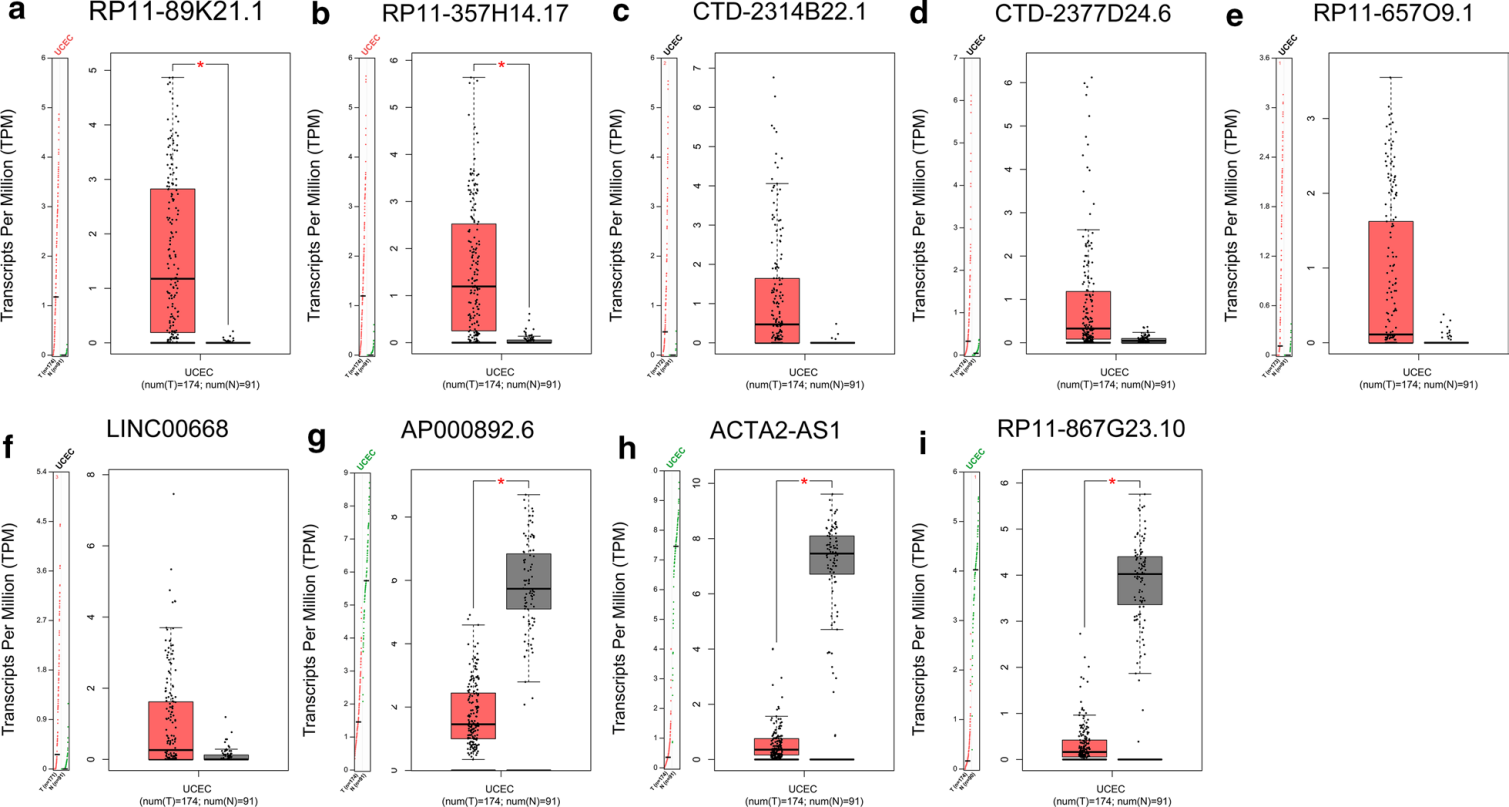
Expression levels of dysregulated lncRNAs in patients with UCEC validated with GEPIA. **a**, **b** RP11-89K21.1 and RP11-357H14.17 were significantly upregulated validated with GEPIA (both *P *< 0.05). **c–f** The expression levels of CTD-2314B22.1, CTD-2377D24.6, RP11-657O9.1 and LINC00668 in UCEC (all *P *> 0.05). **g–i** AP000892.6, ACTA2-AS1, and RP11-867G23.10 were significantly downregulated in UCEC validated with GEPIA (all *P *< 0.05). TPM:Transcripts per Million

To investigated the roles of lncRNAs in the tumorigenesis, development of UCEC, we firstly identified differentially expressed lncRNAs in the LncRNA-TCGA module of cirlncRNAnet. A total of 10,978 lncRNAs were detected in Uterine Corpus Endometrial Carcinoma (UCEC), of which 121 lncRNAs were dysregulated (77 up-regulated and 44 down-regulated) (|Log2 fold change| > 4 and *P *< 0.01) (Additional file 1: Table S1). We further verified the expression of 121 differentially expressed lncRNAs in UCEC using the GEPIA and found that only RP11-89K21.1 and RP11-357H14.17 were significantly overexpressed in UCEC (*P *< 0.05) (Fig. 1a, b), consistent with the results from cirlncRNAnet. The expression of CTD-2314B22 0.1, CTD-2377D24.6, RP11-657O9.1 and LINC00668 were higher in UCEC than those in normal tissues, but the differences were not statistically significant (*P *> 0.05) (Fig. 1c–f). The expression of AP000892.6, ACTA2-AS1 and RP11-867G23.10 was significantly decreased in UCEC compared with normal tissues (*P* < 0.05) (Fig. 1h–i).

### Prognostic values of dysregulated lncRNAs including RP11-89K21.1 and RP11-357H14.17 in UCEC analyzed by GEPIA

**Fig. 2:**
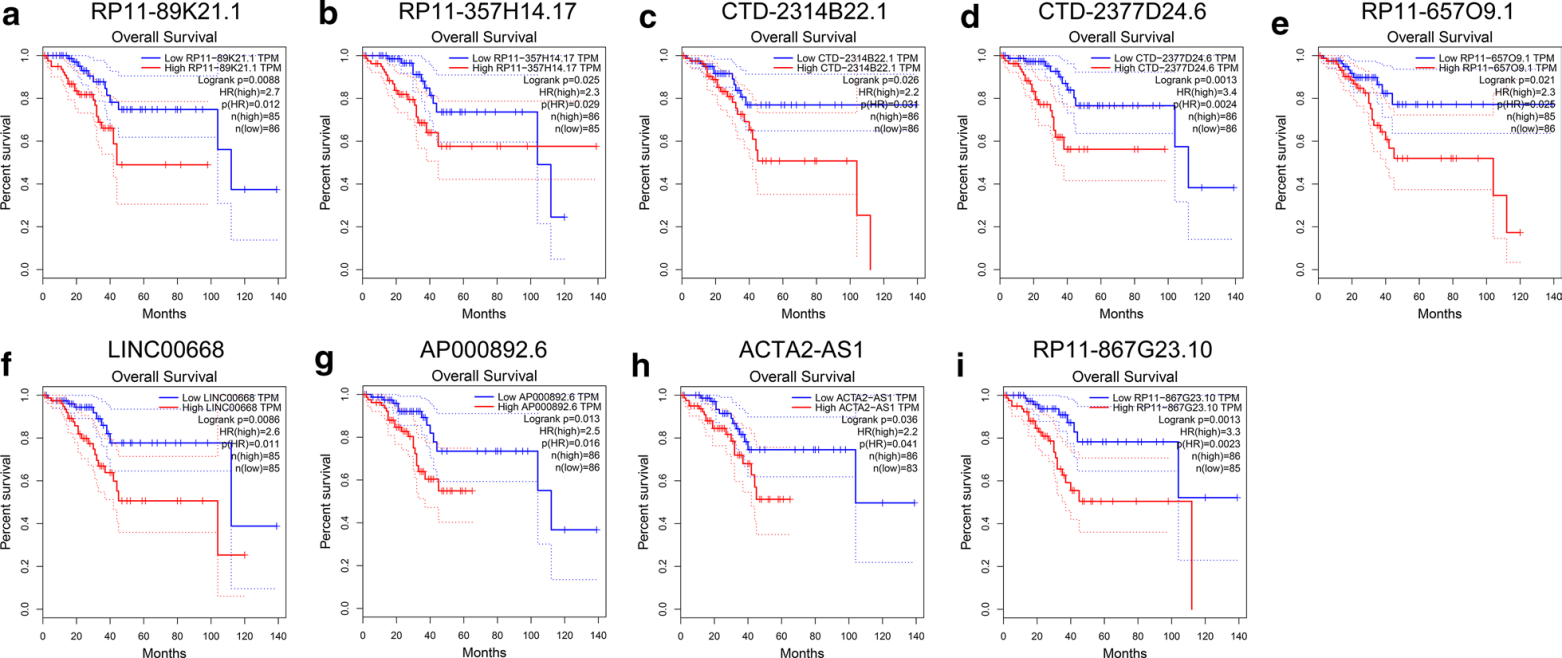
Prognostic values of dysregulated lncRNAs including RP11-89K21.1 and RP11-357H14.17 in UCEC analyzed by GEPIA. **a–i** Relationship between RP11-89K21.1 (**a**), RP11-357H14.17 (**b**), CTD-2314B22.1 (**c**), CTD-2377D24.6 (**d**), RP11-657O9.1 (**e**), LINC00668 (**f**), AP000892.6 (**g**), ACTA2-AS1 (**h**), RP11-867G23.10 (**i**) and overall survival (OS) of patients with UCEC

We further explored the prognostic values of the 121 differentially expressed lncRNAs in UCEC using GEPIA. The results showed that high expressions of RP11-89K21.1, RP11-357H14.17, CTD-2314B22.1, CTD-2377D24.6, RP11-657O9.1 and LINC00668 were significantly correlated with shortened overall survival (OS) in UCEC (*P *= 0.0088, *P *= 0.025, *P *= 0.026, *P *= 0.0013, *P *= 0.021, and *P *= 0.0086, respectively) (Fig. 2a–f, Table 1). Additionally, the expressions of AP000892.6, ACTA2-AS1 and RP11-867G23.10 were decreased in UCEC, while high expressions of AP000892.6, ACTA2-AS1 and RP11-867G23.10 were significantly associated with poor prognosis (all *P *< 0.05) (Fig. 2g–i). Kaplan–Meier plotter showed that high expressions of RP11-89K21.1 (Additional file 1: Figure S1a), LINC00668 (Additional file 1: Figure S1d) and ACTA2-AS1 (Additional file 1: Figure S1e) were also correlated with OS in UCEC (Additional file 1: Table S1), RP11-357H14.17, CTD-2377D24.6 and AP000892.6 were not detected in Kaplan–Meier plotter. The above results suggest that high expression of RP11-89K21.1 and RP11-357H14.17 may play important roles in the occurrence, development and prognosis of UCEC.

**Table 1:** The expression levels and prognostic values of differentially expressed lncRNAs including RP11-89K21.1 and RP11-357H14.17 in UCEC

Gene	Ensembl ID	Genome location	Log2 fold change	*P* adj	Overall survival
RP11-89K21.1	ENSG00000259439	chr2:44921077-44939199	7.113667606	3.31E − 32	0.0088
RP11-357H14.17	ENSG00000272763	chr17:48635923-48647023	5.64219224	3.26E − 28	0.025
CTD-2314B22.1	ENSG00000258314	chr14:19054341-19055551	5.415117641	5.14E − 11	0.026
CTD-2377D24.6	ENSG00000244649	chr17:48705203-48707346	5.381493576	4.72E − 21	0.0013
RP11-657O9.1	ENSG00000240086	chr3:135373795-135439822	4.629555176	1.49E − 09	0.021
LINC00668	ENSG00000265933	chr18:6925478-6928572	4.089425104	1.05E − 11	0.0086
AP000892.6	ENSG00000280143	chr11:117204967-117210292	− 4.08259009	1.28E − 36	0.013
ACTA2-AS1	ENSG00000180139	chr10:88932390-88933838	− 4.164362472	2.49E − 36	0.036
RP11-867G23.10	ENSG00000254510	chr11:66409194-66417137	− 4.737563254	1.01E − 29	0.0013

### The cellular location and co-expressed genes of RP11-89K21.1 and RP11-357H14.17

**Fig. 3:**
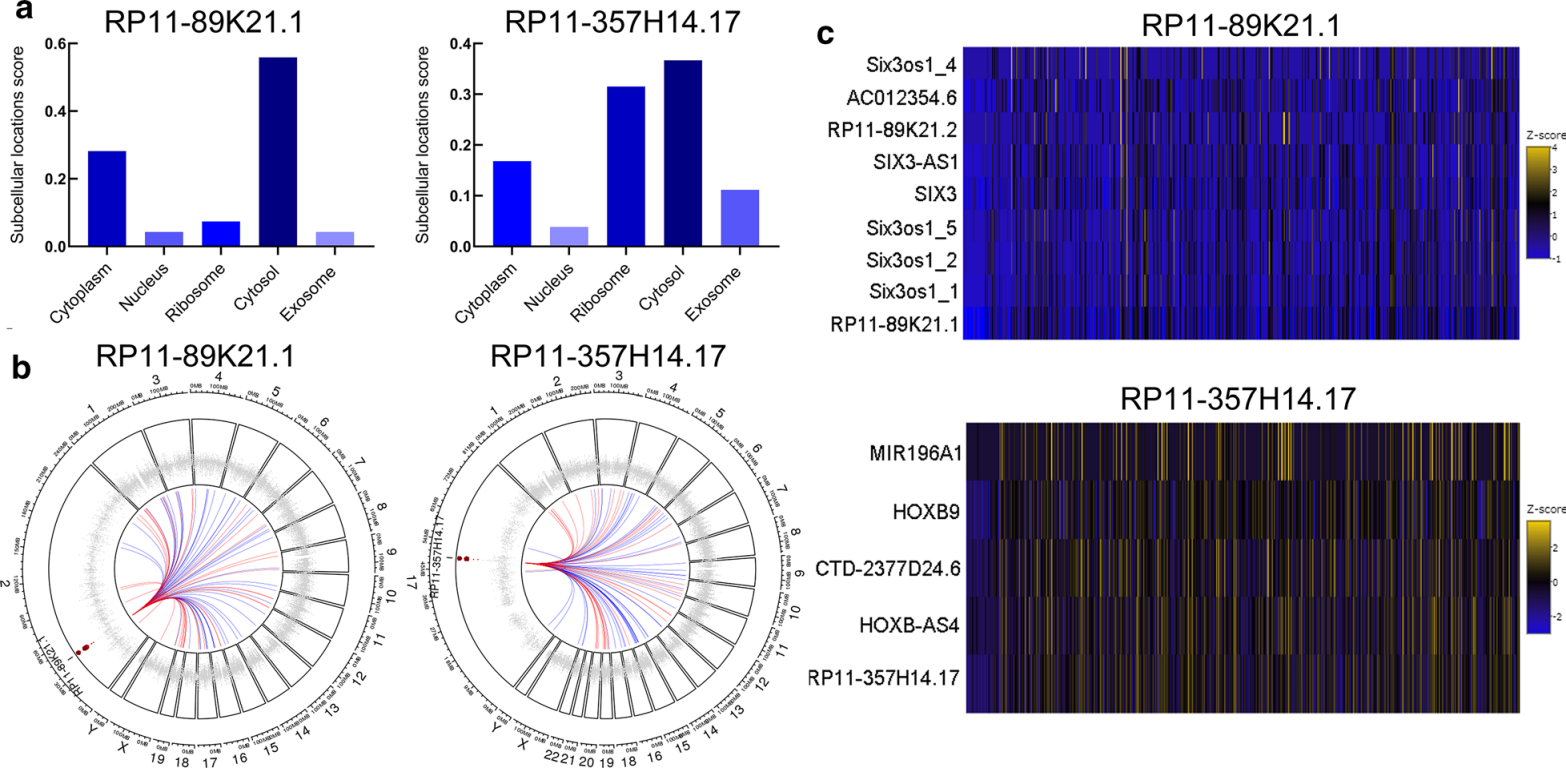
The cellular location and co-expression genes of RP11-89K21.1 and RP11-357H14.17 in UCEC. **a** Subcellular localizations of RP11-89K21.1 and RP11-357H14.17 in UCEC indicated by lncLocator. **b** Genome-wide distribution of the co-expressed genes correlated with the RP11-89K21.1 and RP11-357H14.17 in UCEC showed by Circos maps with circlncRNAnet (top 50). Red: genes with positive correlation; blue: genes with negative correlation; small dots: genes enrichment in human chromosome. **c** The genes co-expressed with RP11-89K21.1 and RP11-357H14.17 in UCEC showed by heat maps with circlncRNAnet (Pearson’s correlation coefficient: |r| > 0.5, *P*-value < 0.05). Z-score: standard score

The cellular localization of lncRNAs played crucial roles in their functions and molecular mechanisms, we explored the subcellular localizations of RP11-89K21.1 and RP11-357H14.17 with lncLocator. The results showed that RP11-89K21.17 was mainly located in cytosol and cytoplasm (score: 0.56 and 0.28, respectively), RP11-357H14.17 was mainly located in cytosol and ribosome (score: 0.37 and 0.32, respectively) (Fig. 3a). Therefore, it was more likely that RP11-89K21.1 and RP11-357H14.17 exerted their biological functions and potential mechanisms through the ceRNA network. We further explored the co-expression gene of RP11-89K21.1 and RP11-357H14.17 and visualized by Circos map and heat map with circlncRNAnet. The circos maps showed that chromosome distribution of the top 50 co-expressed genes associated with RP11-89K21.1 and RP11-357H14.17, which were mainly localized in the autosomes. There was no particular enrichment in chromosome 2 (where RP11-89K21.1 locates) and 7 (where RP11-357H14.17 locates). The heat map indicated that AC012354.6, RP11-89K21.2, SIX3, SIX3-AS1, and Six3os1_1/2/4/5 were the co-expression gene of RP11-89K21.1, CTD-2377D24.6, HOXB-AS4, HOXB9 and MIR196A1 were the co-expression gene of RP11-357H14.17 (all *P *< 0.05) (Fig. 3b, c, Additional files 2, 3).

### Transcriptional regulation and protein interaction of RP11-89K21.1 and RP11-357H14.17

**Fig. 4:**
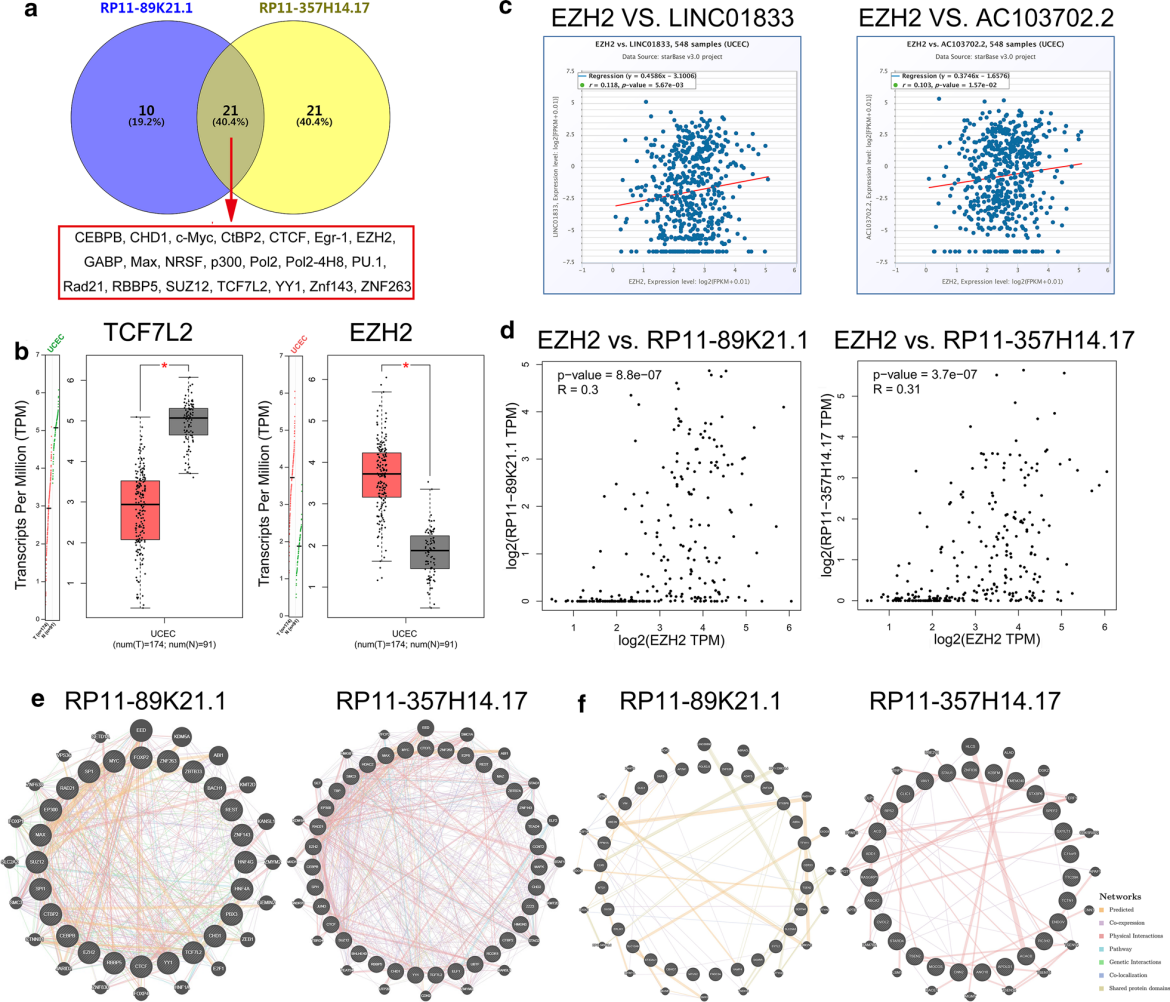
Transcriptional regulation and protein interaction of RP11-89K21.1 and RP11-357H14.17. **a** The common transcriptional factors of RP11-89K21.1 and RP11-357H14.17 showed by Venny. **b** The expression levels of TCF7L2 and EZH2 by GEPIA. **c** The correlation between EZH2 and RP11-89K21.1, RP11-357H14.17 showed by scatter plot with Starbase. **d** The correlation between EZH2 and RP11-89K21.1, RP11-357H14.17 further displayed by scatter plot with GEPIA. **e** The network of transcriptional regulations of RP11-89K21.1 and RP11-357H14.17 with GeneMANIA. **f** The network of protein interactions of RP11-89K21.1 and RP11-357H14.17 with GeneMANIA, respectively. TPM: Transcripts per Million; LINC01833: the alternative gene name of RP11-89K21.1; AC103702.2: the alternative gene name of RP11-357H14.17

Furthermore, we explored the transcriptional factors (TFs) and binding proteins of RP11-89K21.1 and RP11-357H14.17 by AnnoLnc. 31 TFs and 42 TFs were identified to be correlated with RP11-89K21.1 and RP11-357H14.17, respectively. 21 TFs of these two lncRNAs were commonly existed in the database (CEBPB, CHD1, c-Myc, CtBP2, CTCF, Egr-1, EZH2, GABP, Max, NRSF, p300, Pol2, Pol2-4H8, PU.1, Rad21, RBBP5, SUZ12, TCF7L2, YY1, Znf143, ZNF263) (Fig. 4a). We found that the expression of EZH2 in UCEC was significantly overexpressed, while the expression of TCF7L2 in UCEC was significantly decreased (both *P *< 0.05). There was no significant difference in the expression levels of other TFs in UCEC (*P *> 0.05) (Fig. 4b). We further investigated that EZH2 expression was positively correlated with both RP11-89K21.1 and RP11-357H14.17 using starBase (r = 0.118, *P *= 5.67e-03 and r = 0.103, *P *= 1.57e-02, respectively) (Fig. 4c). Based on the correlation coefficients were too small, we further explored the correlation between EZH2 and RP11-89K21.1, RP11-357H14.17 using GEPIA, the result showed that the expression of EZH2 was positively correlated with both RP11-89K21.1 (R = 0.3, *P *= 8.8e-07) and RP11-357H14.17 (R = 0.31, *P *= 3.7e-07) (Fig. 4d). What’s more, the binding proteins of RP11-89K21.1 included CBWD7, GUSB, ABRA, MTUS2, VAMP4 (top 5) (Fig. 4e), and the binding proteins of RP11-357H14.17 included CLIC1, CNN2, TCTN1, TMEM240, ZNF836 (top 5) (Fig. 4f). The networks of the transcription factors (Fig. 4e) and binding proteins (Fig. 4f) were visualized through GeneMANIA (Additional files: 4, 5, 6, 7).

### Construction of lncRNA–miRNA–mRNA network

**Fig. 5:**
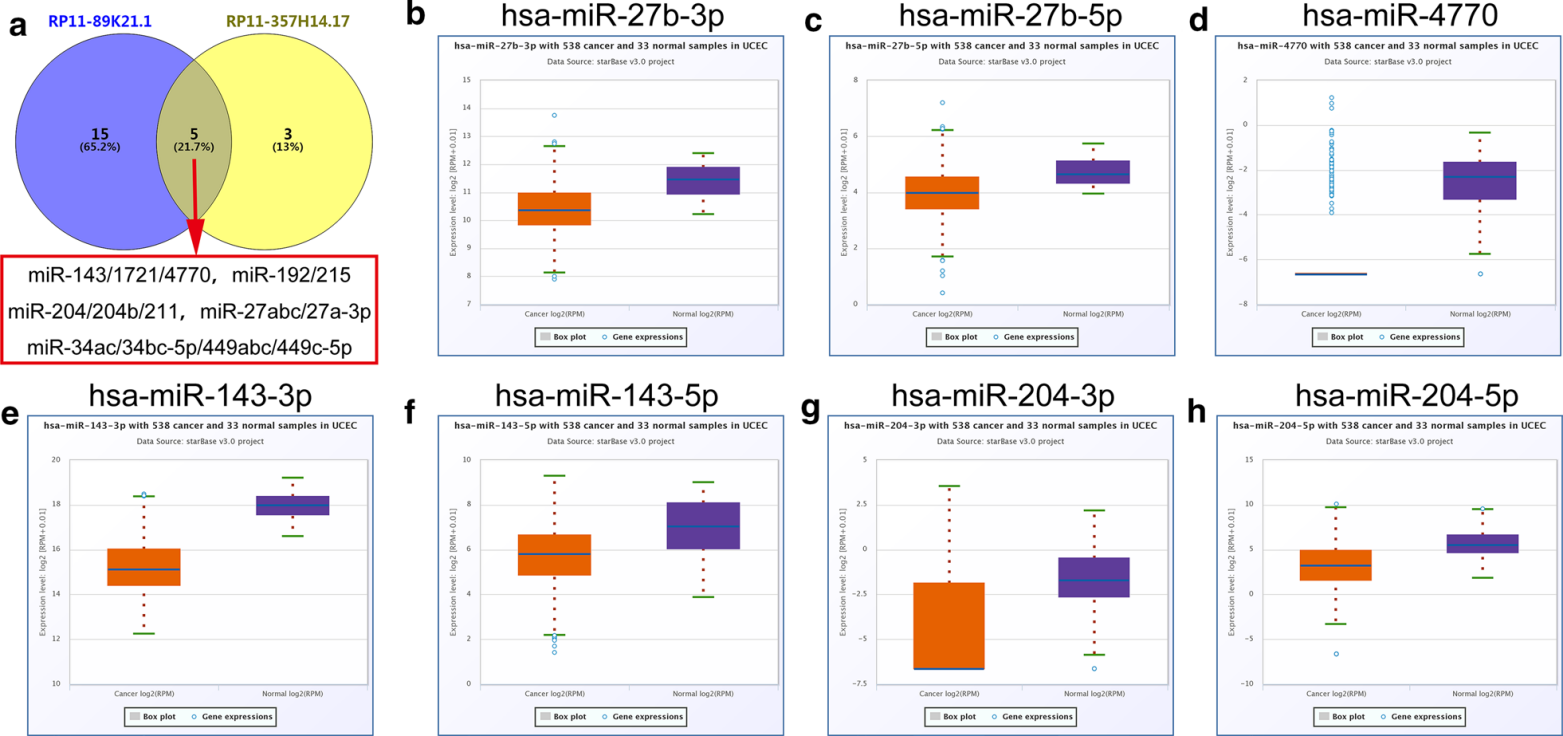
The expression of common binding miRNAs of RP11-89K21.1 and RP11-357H14.17 in UCEC with starBase. **a** The common binding miRNAs of RP11-89K21.1 and RP11-357H14.17 showed by Venny. **b–h** the expression levels of miR-27b (**b, c**), miR-4770 (**d**), miR-143 (**e**, **f**), miR-204 (**g, h**) were downregulated in UCEC compared with normal tissues (*P *< 0.05)

**Fig. 6:**
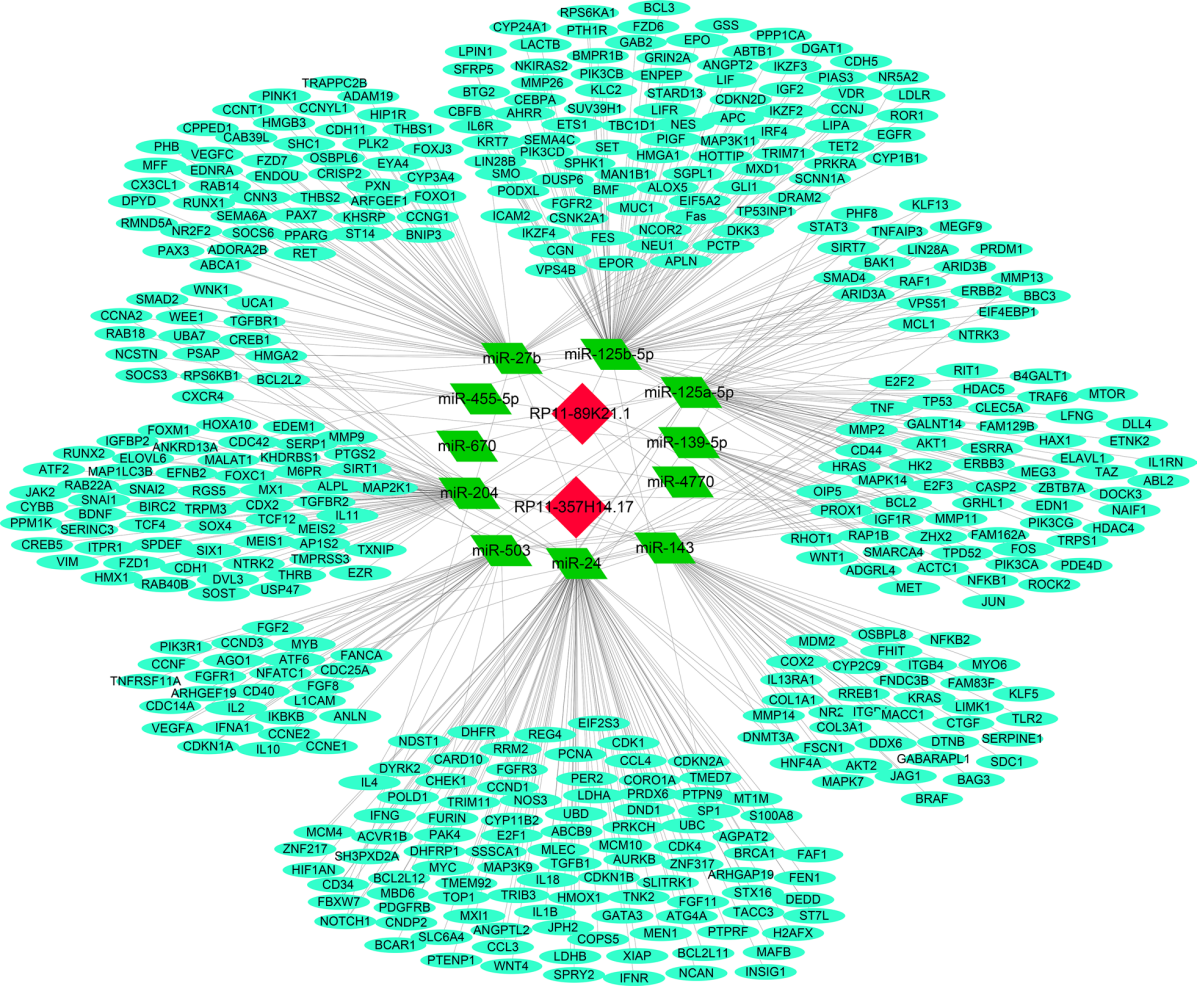
Construction of lncRNA-miRNA-mRNA regulatory network. The binding miRNAs of RP11-89K21.1 and RP11-357H14.17 were predicted by AnnoLnc, and the targets of these miRNAs were retrieved from the miRTarBase. The regulatory network of lncRNA-miRNA-mRNA was visualized using Cytoscape 3.7.1, including 2 lncRNAs (RP11-89K21.1 and RP11-357H14.17), 11 miRNAs and 183 target genes

The subcellular locations of lncRNAs were closely correlated with their potential functions and mechanisms in tumors. We found that both RP11-89K21.1 and RP11-357H14.17 were mainly located in cytosol, and it was possible that RP11-89K21.1 and RP11-357H14.17 achieved their biological functions through the ceRNA mechanism. Thus, the binding miRNAs of RP11-89K21.1 and RP11-357H14.17 were predicted by AnnoLnc. The expressions of potential miRNAs were further validated by starBase. The results showed that 22 miRNAs families and 10 miRNAs families were associated with RP11-89K21.1 and RP11-357H14.17, respectively. There were 5 miRNAs families overlapped in the lncRNAs binding miRNAs (Fig. 5a, Table 2 and Additional file 1: Table S2), of which the expression of miR-27b, miR-4770, miR-143, miR-204 in UCEC was significantly decreased (all *P *< 0.05) (Fig. 5b–h). The expression of miR-125a-5p, miR-125b-5p, miR-139-5p, miR-670-3p, miR-24-1-5p, miR-503 in UCEC was also significantly decreased (all *P *< 0.05) (Additional file 1: Figure S2, Table S2). For RP11-89K21.1, miR-27b, miR-4770, miR-143, miR-204, miR-125a-5p, miR-125b-5p, miR-139-5p and miR-670-3p were regarded as candidate miRNAs. For RP11-357H14.17, miR-27b, miR-4770, miR-143, miR-204, miR-24-1-5p and miR-503 were considered as candidate miRNAs. The targeted mRNAs of the candidate miRNAs were further predicted by miRTarBase. As shown in Fig. 6, lncRNA-miRNA-mRNA regulatory network was constructed by Cytoscape, there were 2 lncRNAs (RP11-89K21.1 and RP11-357H14.17), 11 miRNAs and 183 target genes included in the interaction network (Fig. 6).

**Table 2:** MiRNAs correlated with RP11-89K21.1 and RP11-357H14.17 predicted with AnnoLnc

LncRNA	MiRNA families
RP11-89K21.1	miR-122/122a/1352
	miR-125a-5p/125b-5p/351/670/4319
	miR-125a-5p/125b-5p/351/670/4319
	miR-128/128ab
	miR-139-5p
	miR-143/1721/4770
	miR-146ac/146b-5p
	miR-146ac/146b-5p
	miR-150/5127
	miR-192/215
	miR-194
	miR-1ab/206/613
	miR-200bc/429/548a
	miR-204/204b/211
	miR-21/590-5p
	miR-216b/216b-5p
	miR-218/218a
	miR-27abc/27a-3p
	miR-34ac/34bc-5p/449abc/449c-5p
	miR-455-5p
	miR-490-3p
	miR-7/7ab
RP11-357H14.17	miR-204/204b/211
	miR-27abc/27a-3p
	miR-192/215
	miR-138/138ab
	miR-34ac/34bc-5p/449abc/449c-5p
	miR-34ac/34bc-5p/449abc/449c-5p
	miR-143/1721/4770
	miR-24/24ab/24-3p
	miR-503
	miR-138/138ab

### Functional enrichment analysis of lncRNA-related targets

**Fig. 7:**
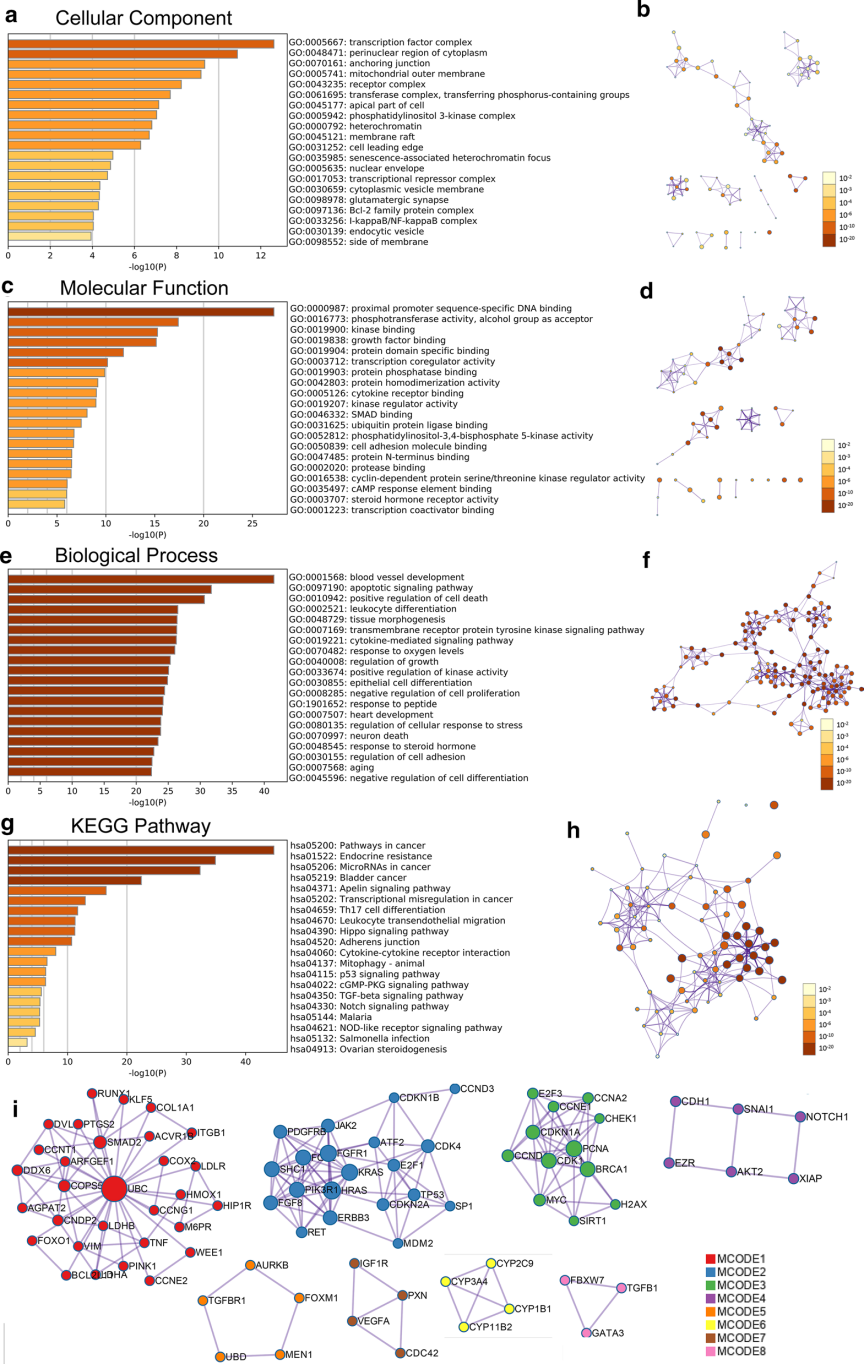
Significant enrichment analysis of GO and KEGG pathways of target genes correlated with RP11-89K21.1 in UCEC with Metascape. a-f Cellular component (**a, b**), molecular function (**c, d**) and biological process (**e, f**) enrichment analysis of RP11-89K21.1-related targets colored by *P*-value with bar graph and network (Top 20). **g, h** KEGG pathways enrichment analysis of RP11-89K21.1-related targets colored by *P*-value with bar graph and network (Top 20). **i** The PPI network formed by protein–protein interaction (PPI) network and the ten most significant MCODE components of RP11-89K21.1 target genes. *GO* Gene Ontology, *KEGG* Kyoto Encyclopedia of Genes and Genomes, *MCODE* Molecular Complex Detection. Above results were colored by *P*-value, where terms containing more genes tend to have a more significant *P*-value

To explore the potential functions and mechanisms of RP11-89K21.1 and RP11-357H14.17 in the development of UCEC, GO and KEGG enrichment analysis of RP11-89K21.1 and RP11-357H14.17 targeted genes were analyzed by Metascape. GO showed that RP11-89K21.1 targeted genes primarily participated in transcription factor complex and perinuclear region of cytoplasm, also regulated proximal promoter sequence-specific DNA binding, phosphotransferase activity, kinase and growth factor binding (Fig. 7a–d, Additional file 1: Table S3–4). The biological processes of RP11-89K21.1 targeted genes mainly involved in blood vessel development, regulation of cell death and differentiation, tissue morphogenesis and response to growth factor (Fig. 7e–f and Table 3). RP11-357H14.17 targeted genes were mainly located in perinuclear region of cytoplasm, participated in cyclin-dependent protein kinase holoenzyme complex, adherens junction and transcription factor complex, also regulated protein kinase activity.

**Table 3:** Significantly enriched GO annotations (Biological Processes) of RP11-89K21.1 in endometrial carcinoma with Metascape (Top 20)

GO	Category	Description	Count	%	Log10 (P)	Log10 (q)
GO:0001568	GO Biological Processes	Blood vessel development	76	9.78	− 41.51	− 37.31
GO:0097190	GO Biological Processes	Apoptotic signaling pathway	59	9.72	− 31.73	− 28.00
GO:0010942	GO Biological Processes	Positive regulation of cell death	63	8.53	− 30.65	− 27.15
GO:0002521	GO Biological Processes	Leukocyte differentiation	50	9.58	− 26.49	− 23.19
GO:0048729	GO Biological Processes	Tissue morphogenesis	56	8.24	− 26.38	− 23.18
GO:0007169	GO Biological Processes	Transmembrane receptor protein tyrosine kinase signaling pathway	58	7.87	− 26.32	− 23.17
GO:0019221	GO Biological Processes	Cytokine-mediated signaling pathway	60	7.54	− 26.26	− 23.14
GO:0070482	GO Biological Processes	Response to oxygen levels	44	11.17	− 26.03	− 22.98
GO:0040008	GO Biological Processes	Regulation of growth	55	8.02	− 25.32	− 22.42
GO:0033674	GO Biological Processes	Positive regulation of kinase activity	52	8.48	− 25.06	− 22.18
GO:0030855	GO Biological Processes	Epithelial cell differentiation	58	7.38	− 24.89	-22.03
GO:0008285	GO Biological Processes	Negative regulation of cell proliferation	57	7.37	− 24.43	− 21.59
GO:1901652	GO Biological Processes	Response to peptide	48	8.99	− 24.20	− 21.38
GO:0007507	GO Biological Processes	Heart development	50	8.49	− 24.09	− 21.29
GO:0080135	GO Biological Processes	Regulation of cellular response to stress	56	7.32	− 23.84	− 21.07
GO:0070997	GO Biological Processes	Neuron death	40	11.27	− 23.81	− 21.05
GO:0048545	GO Biological Processes	Response to steroid hormone	41	10.62	− 23.41	− 20.67
GO:0030155	GO Biological Processes	Regulation of cell adhesion	53	7.40	− 22.76	− 20.09
GO:0007568	GO Biological Processes	Aging	37	11.60	− 22.47	− 19.84
GO:0045596	GO Biological Processes	Negative regulation of cell differentiation	54	7.11	− 22.38	− 19.76

transcription factor and kinase binding (Fig. 8a–d, Additional file 1: Table S5, 6). RP11-89K21.1 targeted genes mainly involved in biological processes such as blood vessel development, regulation of transferase activity, apoptotic signaling pathway, regulation of cell death and cell differentiation, response to oxygen levels (Fig. 8e–f and Table 4).

**Fig. 8:**
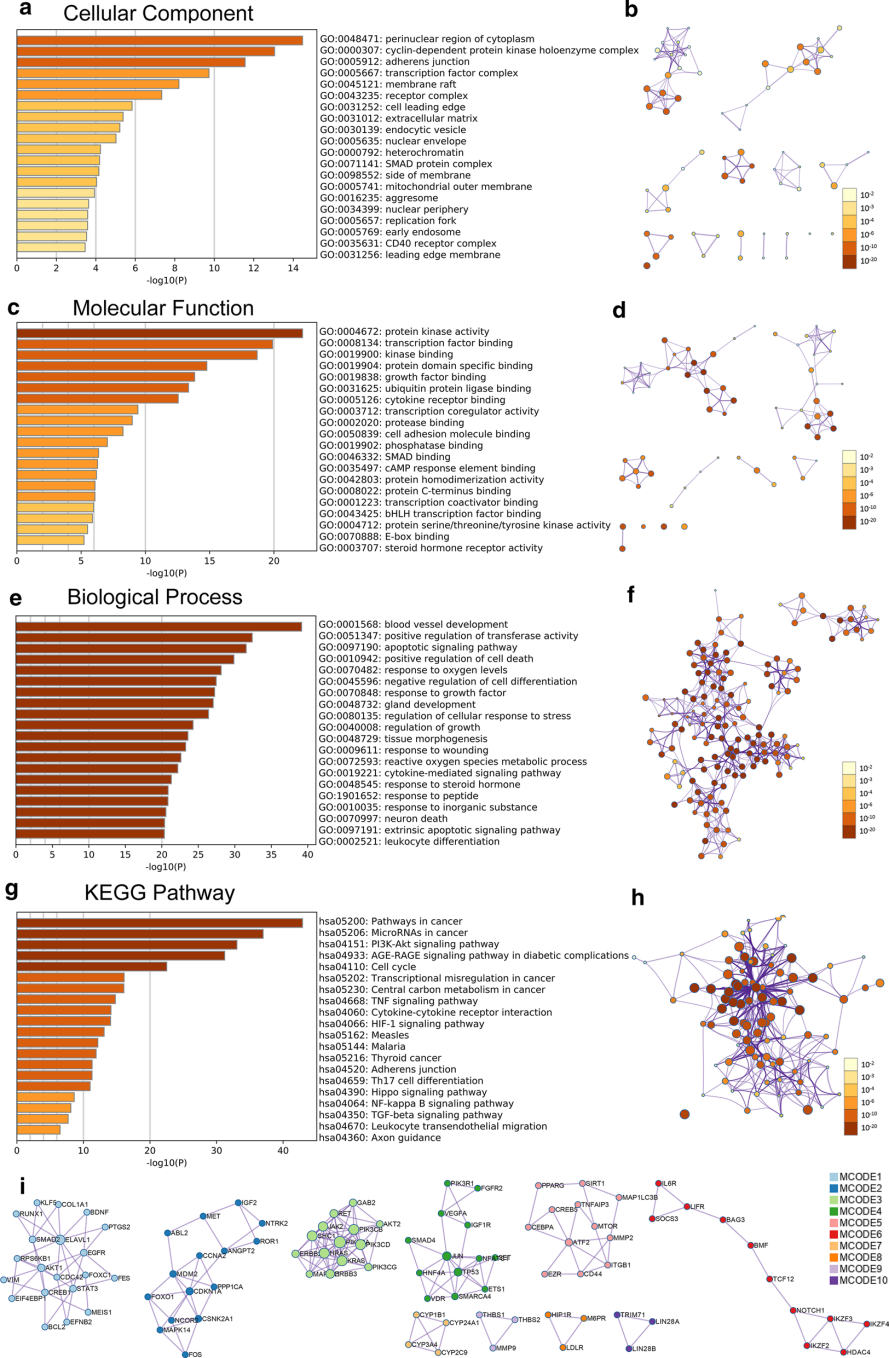
Significant enrichment analysis of GO and KEGG pathways of target genes correlated with RP11-357H14.17 in UCEC with Metascape. **a–f** Cellular component (**a, b**), molecular function (**c, d**) and biological process (**e, f**) enrichment analysis of RP11-357H14.17-ralated targets colored by *P*-value with bar graph and network (Top 20). **g, h** KEGG pathways enrichment analysis of RP11-357H14.17-related targets colored by *P*-value with bar graph and network (Top 20). **i** The PPI network formed by protein–protein interaction (PPI) network and the ten most significant MCODE components of RP11-357H14.17 target genes. GO: Gene Ontology; KEGG: Kyoto Encyclopedia of Genes and Genomes; MCODE: Molecular Complex Detection. Above results were colored by *P*-value, where terms containing more genes tend to have a more significant *P*-value

**Table 4:** Significantly enriched GO annotations (Biological Processes) of RP11-357H14.17 in endometrial carcinoma with Metascape (Top 20)

GO	Category	Description	Count	%	Log10 (P)	Log10 (q)
GO:0001568	GO Biological Processes	Blood vessel development	70	9.01	− 39.16	− 34.96
GO:0051347	GO Biological Processes	Positive regulation of transferase activity	60	8.67	− 32.38	− 28.66
GO:0097190	GO Biological Processes	Apoptotic signaling pathway	56	9.23	− 31.55	− 27.95
GO:0010942	GO Biological Processes	Positive regulation of cell death	59	7.98	− 29.87	− 26.52
GO:0070482	GO Biological Processes	Response to oxygen levels	44	11.17	− 28.12	− 24.97
GO:0045596	GO Biological Processes	Negative regulation of cell differentiation	57	7.51	− 27.45	− 24.42
GO:0070848	GO Biological Processes	Response to growth factor	56	7.61	− 27.24	− 24.24
GO:0048732	GO Biological Processes	Gland development	45	10.18	− 27.04	− 24.07
GO:0080135	GO Biological Processes	Regulation of cellular response to stress	56	7.32	− 26.39	− 23.49
GO:0040008	GO Biological Processes	Regulation of growth	51	7.43	− 24.28	− 21.47
GO:0048729	GO Biological Processes	Tissue morphogenesis	50	7.35	− 23.57	− 20.81
GO:0009611	GO Biological Processes	Response to wounding	50	7.24	− 23.26	− 20.51
GO:0072593	GO Biological Processes	Reactive oxygen species metabolic process	34	11.89	− 22.61	− 19.92
GO:0019221	GO Biological Processes	Cytokine-mediated signaling pathway	52	6.53	− 22.18	− 19.53
GO:0048545	GO Biological Processes	Response to steroid hormone	37	9.59	− 21.30	− 18.68
GO:1901652	GO Biological Processes	Response to peptide	42	7.87	− 20.86	− 18.26
GO:0010035	GO Biological Processes	Response to inorganic substance	43	7.62	− 20.83	− 18.25
GO:0070997	GO Biological Processes	Neuron death	35	9.86	− 20.55	− 18.00
GO:0097191	GO Biological Processes	Extrinsic apoptotic signaling pathway	29	12.95	− 20.37	− 17.82
GO:0002521	GO Biological Processes	Leukocyte differentiation	41	7.85	− 20.34	− 17.80

KEGG enrichment analysis showed that RP11-89K21.1 targeted genes were significantly enriched in pathways in cancer, endocrine resistance and microRNAs in cancer, regulated apelin signaling pathway, Th17 cell differentiation and hippo signaling pathway (Fig. 7g, h, Table 5). RP11-357H14.17 targeted genes were significantly enriched in pathways in cancer, microRNAs in cancer, PI3K-AKT signaling pathway, AGE-RAGE signaling pathway in diabetic complications, cell cycle and cytokine-mediated signaling pathway (Fig. 8g, h, Table 6). These signaling pathways played key roles in the occurrence and development of a variety of tumors, including endometrial carcinoma. Moreover, in order to better understand the relationship between RP11-89K21.1, RP11-357H14.17 and UCEC, we performed protein–protein interaction (PPI) enrichment analysis using Metascape (Figs. 7i, 8i). The most important 10 and 8 MCODE components in PPI network, pathway and enrichment process analysis were applied to each MCODE component independently.

**Table 5:** Significantly enriched KEGG pathway of RP11-89K21.1 in endometrial carcinoma with Metascape (Top 20)

GO	Category	Description	Count	%	Log10 (P)	Log10 (q)
hsa05200	KEGG Pathway	Pathways in cancer	61	15.44	− 44.75	− 42.06
hsa01522	KEGG Pathway	Endocrine resistance	32	33.33	− 34.89	− 32.68
hsa05206	KEGG Pathway	MicroRNAs in cancer	45	15.05	− 32.32	− 30.23
hsa05219	KEGG Pathway	Bladder cancer	18	43.90	− 22.42	− 20.98
hsa04371	KEGG Pathway	Apelin signaling pathway	22	15.94	− 16.49	− 15.42
hsa05202	KEGG Pathway	Transcriptional misregulation in cancer	21	11.67	− 12.98	− 12.02
hsa04659	KEGG Pathway	Th17 cell differentiation	16	14.95	− 11.70	− 10.80
hsa04670	KEGG Pathway	Leukocyte transendothelial migration	16	14.04	− 11.27	− 10.39
hsa04390	KEGG Pathway	Hippo signaling pathway	18	11.69	− 11.22	− 10.36
hsa04520	KEGG Pathway	Adherens junction	13	18.06	− 10.69	− 9.84
hsa04060	KEGG Pathway	Cytokine-cytokine receptor interaction	19	7.04	− 8.02	− 7.26
hsa04137	KEGG Pathway	Mitophagy-animal	9	13.85	− 6.54	− 5.82
hsa04115	KEGG Pathway	p53 signaling pathway	9	13.04	− 6.31	− 5.60
hsa04022	KEGG Pathway	cGMP-PKG signaling pathway	13	7.98	− 6.28	− 5.58
hsa04350	KEGG Pathway	TGF-beta signaling pathway	9	10.71	− 5.58	− 4.90
hsa04330	KEGG Pathway	Notch signaling pathway	7	14.58	− 5.36	− 4.69
hsa05144	KEGG Pathway	Malaria	7	14.29	− 5.30	− 4.64
hsa04621	KEGG Pathway	NOD-like receptor signaling pathway	12	7.06	− 5.29	− 4.64
hsa05132	KEGG Pathway	Salmonella infection	8	9.30	− 4.56	− 3.94
hsa04913	KEGG Pathway	Ovarian steroidogenesis	5	10.00	− 3.19	− 2.61

**Table 6:** Significantly enriched KEGG pathway of RP11-357H14.17 in endometrial carcinoma with Metascape (Top 20)

GO	Category	Description	Count	%	Log10 (P)	Log10 (q)
hsa05200	KEGG Pathway	Pathways in cancer	57/395	14.43	− 42.91	− 40.22
hsa05206	KEGG Pathway	MicroRNAs in cancer	47/299	15.72	− 36.99	− 34.60
hsa04151	KEGG Pathway	PI3K-Akt signaling pathway	46/342	13.45	− 33.06	− 30.84
hsa04933	KEGG Pathway	AGE-RAGE signaling pathway in diabetic complications	29/99	29.29	− 31.22	− 29.22
hsa04110	KEGG Pathway	Cell cycle	25/124	20.16	− 22.51	− 21.02
hsa05202	KEGG Pathway	Transcriptional misregulation in cancer	23/180	12.78	− 16.11	− 14.80
hsa05230	KEGG Pathway	Central carbon metabolism in cancer	16/65	24.62	− 16.07	− 14.78
hsa04668	KEGG Pathway	TNF signaling pathway	18/108	16.67	− 14.80	− 13.55
hsa04060	KEGG Pathway	Cytokine-cytokine receptor interaction	25/270	9.26	− 14.14	− 12.92
hsa04066	KEGG Pathway	HIF-1 signaling pathway	17/101	16.83	− 14.08	− 12.88
hsa05162	KEGG Pathway	Measles	18/134	13.43	− 13.10	− 11.98
hsa05144	KEGG Pathway	Malaria	12/49	24.49	− 12.16	− 11.06
hsa05216	KEGG Pathway	Thyroid cancer	10/29	34.48	− 11.90	− 10.86
hsa04520	KEGG Pathway	Adherens junction	13/72	18.06	− 11.31	− 10.30
hsa04659	KEGG Pathway	Th17 cell differentiation	15/107	14.02	− 11.29	− 10.29
hsa04390	KEGG Pathway	Hippo signaling pathway	17/154	11.04	− 11.00	− 10.02
hsa04064	KEGG Pathway	NF-kappa B signaling pathway	12/95	12.63	− 8.61	− 7.76
hsa04350	KEGG Pathway	TGF-beta signaling pathway	11/84	13.10	− 8.11	− 7.27
hsa04670	KEGG Pathway	Leukocyte transendothelial migration	12/114	10.53	− 7.70	− 6.86
hsa04360	KEGG Pathway	Axon guidance	13/175	7.43	− 6.49	− 5.73

### Correlation between the expression of RP11-89K21.1, RP11-357H14.17 and immune infiltration with ImmucLnc

In order to investigate the relationship between RP11-89K21.1, RP11-357H14.17 and immune infiltration, ImmucLnc database was employed to detect RP11-89K21.1, RP11-357H14.17 correlated immune cell types (CD8_T cell, Macrophage, Dendritic, BMSCs and CD4_T cell, and Neutrophil). We found that the expression of RP11-89K21.1 was negatively correlated with CD8_T cell (Rs Value = − 0.159, *P *= 0) and Macrophage (Rs Value = − 0.11, *P *= 0.01), and positively correlated with CD4_T cell (Rs Value = 0.106, *P *= 0.013) and Neutrophil (Rs Value = 0.138, *P *= 0.001) (Table 7). The expression of RP11-357H14.17 was negatively correlated with CD8_T cell (Rs Value = − 0.114, *P *= 0.008) and positively correlated with CD4_T cell (Rs Value = 0.102, *P *= 0.017) (Table 8).

**Table 7:** Correlation of RP11-89K21.1 with immune infiltration analyzed by ImmucLnc

Cancer	LncRNA ID	LncRNA symbol	Immune cell	*P* value	Rs value^b^
UCEC	ENSG00000259439	LINC01833^a^	CD8_T cell	0	− 0.159
UCEC	ENSG00000259439	LINC01833	Macrophage	0.01	− 0.11
UCEC	ENSG00000259439	LINC01833	Dendritic	0.981	− 0.001
UCEC	ENSG00000259439	LINC01833	B_cell	0.404	0.036
UCEC	ENSG00000259439	LINC01833	CD4_T cell	0.013	0.106
UCEC	ENSG00000259439	LINC01833	Neutrophil	0.001	0.138

^a^ LINC01833 the alternative gene name of RP11-89K21.1, ^b^ The correlation coefficient

**Table 8:** Correlation of RP11-357H14.17 with immune infiltration analyzed by ImmucLnc

Cancer	LncRNA ID	LncRNA symbol	Immune cell	*P* value	Rs value^a^
UCEC	ENSG00000272763	RP11-357H14.17	CD8_T cell	0.008	− 0.114
UCEC	ENSG00000272763	RP11-357H14.17	Dendritic	0.072	− 0.077
UCEC	ENSG00000272763	RP11-357H14.17	Macrophage	0.101	− 0.07
UCEC	ENSG00000272763	RP11-357H14.17	B_cell	0.279	− 0.046
UCEC	ENSG00000272763	RP11-357H14.17	Neutrophil	0.307	− 0.044
UCEC	ENSG00000272763	RP11-357H14.17	CD4_T cell	0.017	0.102

^a^ The correlation coefficient

## Discussion

A series of biological processes are involved in the occurrence and progression of EC, such as abnormal expression of genes and transcription factors, dysregulation of cellular signal transduction pathway and imbalance of cell microenvironment homeostasis. Pathological changes and molecular characteristics determine the level of risk and prognosis of patients with EC. In recent years, 1ncRNAs have been identified to exert various malignant biological behaviors in EC including differentiation, proliferation, invasion and metastasis [30]. Therefore, it is valuable and helpful to explore the potential functions and molecular mechanisms of lncRNAs in EC, which contribute to prognostic prediction and therapeutic target of endometrial carcinoma.

In this study, 121 differentially expressed lncRNAs in UCEC were identified by circlncRNAnet, including 77 upregulated and 44 downregulated lncRNAs. We further confirmed for the first time that only high expressions of RP11-89K21.1 and RP11-357H14.17 were significantly associated with shortened OS and poor prognosis of patients with UCEC, which suggested that RP11-89K21.1 and RP11-357H14.17 played oncogene roles in the occurrence, progression of endometrial carcinoma. It was reported that the expression of lncRNAs were regulated by transcription factors [31]. We found that EZH2 was the common transcriptional regulator of RP11-89K21.1 and RP11-357H14.17 in endometrial carcinoma with AnnoLnc, Moreover, EZH2 was positively correlated with the expression of RP11-89K21.1 and RP11-357H14.17. Some studies have showed that lncRNA DLEU2 interacted with EZH2 to promote the proliferation, migration and invasion of hepatocellular carcinoma, thus accelerating the malignant progression of hepatocellular carcinoma [32]. In gastric cancer, lncRNA UCA1 enhanced the translation of cyclin D1 via recruiting EZH2 and further precipitated the proliferation and cell cycle progression of gastric cancer [33]. In lung cancer, the expression of lncRNA-SVUGP2 could be suppressed by EZH2 and further promoted the occurrence and development of lung cancer via Wnt/β-catenin pathway [34]. These studies suggest that there exists potential regulatory mechanism between EZH2 and RP11-89K21.1, RP11-357H14.17 involved in the occurrence and development of endometrial carcinoma, and the specific mechanism remains to be further explored and verified.

Studies have shown that lncRNAs are located in different subcellular structures, including cytoplasm, nucleus, ribosome, cytosol and exosome. Functions and regulatory mechanisms of lncRNAs are closely associated with subcellular localization. We detected that RP11-89K21.1 and RP11-357H14.17 were mainly located in cytosol. Growing evidence suggested that, in the cytoplasm and cytosol, lncRNAs not only regulated the stability and translation of mRNA, but also had an impact on the post-transcriptional modification of proteins and cell signal transduction. Based on the ceRNA hypothesis, lncRNA can competitively bind to miRNAs acting as sponge of miRNAs, detaining or adsorbing miRNAs, thus relieving the inhibition of miRNAs on downstream target genes [35, 36]. Some studies have shown that exosomal lncRNA ARSR could competitively bind to miR-34 and miR-449 to regulate the expression of AXL, c-MET, and then promoted the drug resistance of renal cell carcinoma [37]. In prostate cancer, lncRNA TTTY15 acted as a ceRNA and negatively regulated miR-let-7 to promote expression of the target genes (CDK6, FN1) [38]. In recent years, mounting studies have shown that lncRNA, acting as ceRNA, regulated the expression of downstream oncogenes and tumor suppressor genes in endometrial carcinoma through a miRNA regulatory mechanism. In endometrial carcinoma, lncRNA HOTAIR facilitated the expression of NPM1 by negatively regulating miR-646, and thereby promoting the proliferation, migration and invasion of EC cells [39]. Maziveyi et al. reported that lncRNA TUSC7 promoted the expression of SOCS4 (SOCS5) through acing as sponge of miR-616, thus inhibiting the proliferation, migration and invasion of endometrial carcinoma [40]. We further explored the potential role of ceRNA network mechanism regulated by RP11-89K21.1 and RP11-357H14.17 in the progression of EC. The binding miRNAs of RP11-89K21.1 and RP11-357H14.17 were retrieved from miRTarBase and 4 overlapped miRNAs (miR-27b, miR-4770, miR-143, miR-204) were downregulated in UCEC, and other RP11-89K21.1 binding miRNA (miR-125a-5p, miR-125b-5p, miR-139-5p, miR-670-3p) and RP11-357H14.17 binding miRNA (miR-24-1-5p, miR-503) were also decreased in UCEC. miRNAs were involved in a variety of malignant biological behaviors and mechanisms such as proliferation, invasion and migration of tumors. The expression of miR-27b-3, miR-204-5p was decreased in EC, and high expression of miR-27b-3 and miR-204-5p could significantly inhibit the proliferation, migration and invasion of EC cells [41, 42]. The expression of miR-143 and miR-503 were also downregulated in EC, and high expression of miR-503 inhibited the proliferation and cell cycle of EC cells through negatively regulating CCND1 [43, 44]. These findings indicated that RP11-89K21.1 and RP11-357H14.17 may play oncogene roles in endometrial carcinoma by regulating candidate miRNAs and their targeted genes. Therefore, we have successfully constructed a new lncRNA-miRNA-mRNA ceRNA regulatory network associated with the prognosis of patients with EC, and further experiments are required to verify molecular mechanisms in the regulatory network.

Many studies have shown that lncRNAs affects the initiation and development of tumors by regulating a variety of molecular mechanisms and signaling pathways. Researchers observed lncRNA LSINCT5 promoted proliferation, invasion and metastasis of EC cells by regulating HMGA2/Wnt/β-catenin signaling pathway [45]. LncRNA OGFRP1 promoted the malignant progression of endometrial carcinoma by regulating the miR124-3p/SIRT1 axis and activating the PI3K/AKT/GSK-3β pathway [46]. In order to better clarify the biological function, molecular mechanism and regulatory network of RP11-89K21.1 and RP11-357H14.17-related targeting genes in EC, we carried out GO and KEGG enrichment analysis with Metascape. It was found that RP11-89K21.1 and RP11-357H14.17-related targeting genes were mainly involved in vasculature development, tissue morphogenesis, cell growth and differentiation, regulation of protein kinase activity and cellular response to stress. KEGG enrichment analysis showed that RP11-89K21.1 and RP11-357H14.17-related targeting genes were significantly enriched in microRNAs in cancer, cytokine-mediated signaling pathway, transmembrane receptor protein tyrosine kinase signaling pathway, apoptotic signaling pathway. The above signaling pathways were closely associated the progression and biological behaviors of EC [47]. Therefore, we speculated that RP11-89K21.1 and RP11-357H14.17 can affect the occurrence, development and biological behavior of EC by regulating the above tumor-related pathways, which provided more evidences for further exploring the molecular mechanisms of RP11-89K21.1 and RP11-357H14.17 in endometrial carcinoma.

Accumulating studies have demonstrated that a large number of immune cells and cytokines can be observed in EC, which can enhance the endogenous anti-tumor immune response and affect prognostic value and immunotherapy of EC. Immunotherapy plays a well-established role in the treatment of EC. Our results showed that the expression of RP11-89K21.1 was negatively correlated with CD8_T cell, Macrophage and positively correlated with CD4_T cell, Neutrophil. The expression of RP11-357H14.17 was negatively correlated with CD8_Tcell and positively correlated with CD4_Tcell. Studies have shown that CD8_T cell and Macrophage could participate in the malignant progression of EC and serve as a potential therapeutic target for EC [48]. CD4_T cell could promote the capacity of initiating CD4_T cell rapidly by mediating immune response, and kill tumor cells directly or indirectly by stimulating and recruiting CD8_T cell cells or other immune cells [49]. The number of CD4_T cells in peripheral blood of patients with EC was significantly increased [50]. Neutrophil was the main type of immune cells in tumors, which can eliminate pathogens and prevent host from being infected by microorganisms, playing a key role in chemotherapy resistance and anti-angiogenesis therapy of tumors [51]. Some studies have displayed that neutrophils were closely correlated with survival and prognosis of patients with EC [52]. The results showed that the above immune cells were of great value in the occurrence, development and prognosis of EC. However, it’s not sufficient to conclude that RP11-89K21.1 and RP11-357H14.17 play critical role in regulating the infiltration of immune cells in tumor microenvironment because of the small correlation coefficients (Rs value), and further experiments are required to verify the function of RP11-89K21.1 and RP11-357H14.17 in immune infiltration.

## Conclusion

In summary, with a series of integrated databases, we demonstrated for the first time that high expressions of RP11-89K21.1 and RP11-357H14.17 were closely associated with the poor prognosis of patients with EC. We further identified transcriptional regulatory factors, co-expressed genes, interacting proteins of RP11-89K21.1 and RP11-357H14.17. Regulatory networks of biological function, signaling pathways may contribute to illuminate the potential function and mechanism of RP11-89K21.1 and RP11-357H14.17 in EC. Moreover, we speculated that the ceRNA network associated with RP11-89K21.1 and RP11-357H14.17 provided novel and valuable insights into the molecular mechanisms underlying the initiation and progression of EC. Therefore, RP11-89K21.1 and RP11-357H14.17 can potentially be identified as tumor biomarkers for early diagnosis, prognosis evaluation and therapeutic targets of EC. Although existing research may not be optimal, we think it should be adequate to make a conclusion that RP11-89K21.1 and RP11-357H14.17 contributed to poor prognosis in EC. The functional experiments of RP11-89K21.1 and RP11-357H14.17 will be further conducted in the follow-up study.

## Supplementary information

**Additional file 1.** The expression, prognosis, predicting miRNAs and functional analysis of RP11-89K21.1 and RP11-357H14.17

**Additional file 2.** Co-expressed genes correlated with RP11-89K21.1

**Additional file 3.** Co-expressed genes correlated with RP11-357H14.17

**Additional file 4.** The binding proteins associated with RP11-89K21.1

**Additional file 5.** The transcription factors correlated with RP11-89K21.1

**Additional file 6.** The binding proteins associated with RP11-357H14.17

**Additional file 7.** The transcription factors correlated with RP11-357H14.17

## Data Availability

The datasets used or analyzed during the current study are available from the corresponding author upon reasonable request.

## Abbreviations

EC: Endometrial carcinoma
lncRNAs: Long noncoding RNAs
UCEC: The Uterine Corpus Endometrial Carcinoma
TCGA: The Cancer Genome Atlas
ceRNA: Competing endogenous RNA
OS: Overall survival
TF: Transcriptional factor
PPI: Protein-protein interaction
GO: Gene Ontology
KEGG: Kyoto Encyclopedia of Genes and Genomes
MCODE: Molecular Complex Detection
EZH2: Enhancer of zeste 2 polycomb repressive complex 2 subunit
TPM: Transcripts per Million
