# Supplementary material for: Identification of long noncoding RNA RP11-89K21.1 and RP11-357H14.17 as prognostic signature of endometrial carcinoma via integrated bioinformatics analysis
Source: Cancer Cell Int. 2020 Jun 24;20:268. doi: 10.1186/s12935-020-01359-9 (PMC7313119; doi:10.1186/s12935-020-01359-9)
Supplement: Supplementary file 1 — Additional file 1. The expression, prognosis, predicting miRNAs and functional analysis of RP11-89K21.1 and RP11-357H14.17 [file 12935_2020_1359_MOESM1_ESM.doc]

**Figure S1 The overall survival of dysregulated lncRNAs including RP11-89K21.1 and RP11-357H14.17 in UCEC analyzed by Kaplan Meier plotter.**

**a-g** Relationship between RP11-89K21.1(**a**), CTD-2314B22.1(**b**), RP11-657O9.1 (**c**), LINC00668 (**d**), ACTA2-AS1(**e**), RP11-867G23.10 (**f**) and overall survival (OS) of patients with UCEC. OS: overall survival; UCEC: Uterine Corpus Endometrial Carcinoma.

**
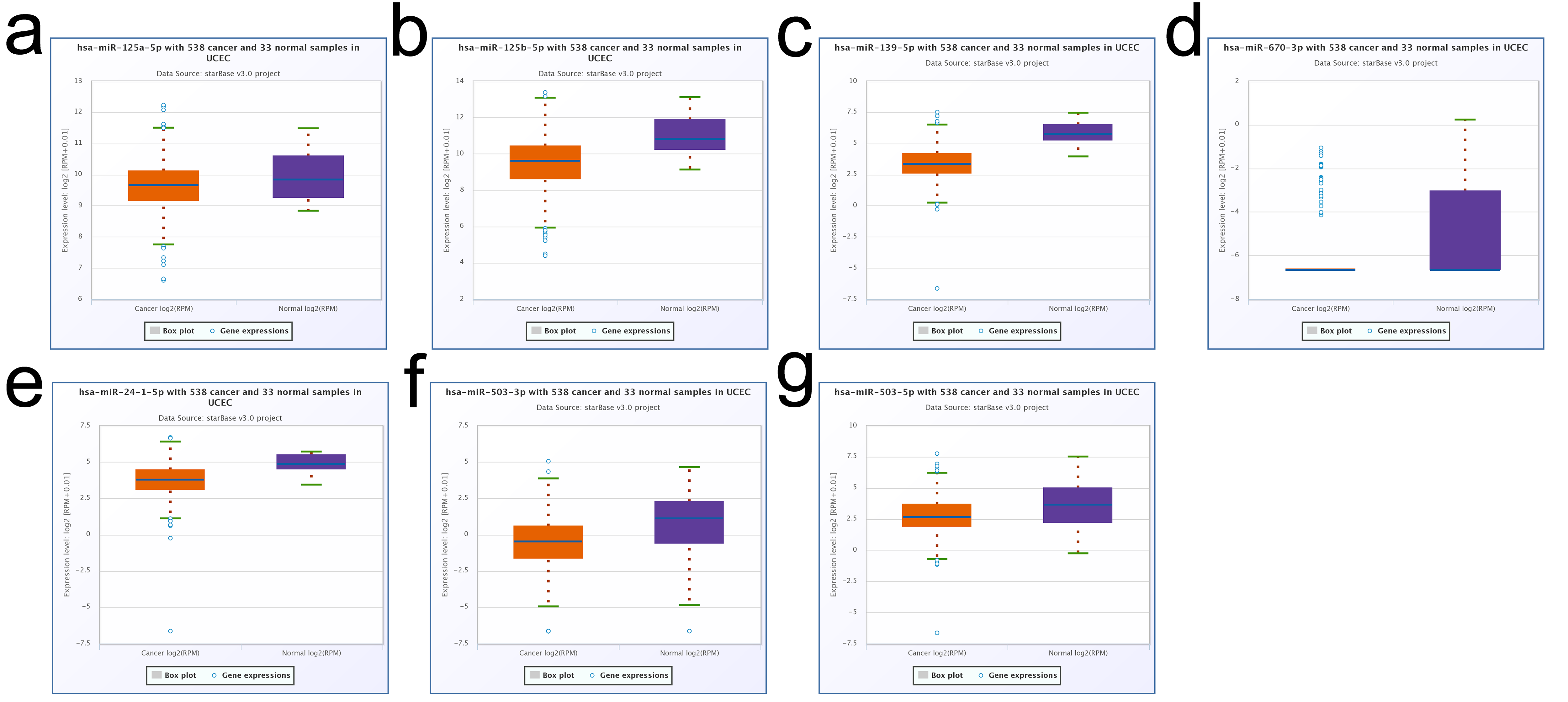
**

**Figure S2 The expression of binding miRNAs of RP11-89K21.1 and RP11-357H14.17 in UCEC with starBase.**

**a-d** The expression levels of miR-125a-5p (**a**), miR-125b-5p (**b**), miR-139-5p (**c**), miR-670-3p (**d**) correlated with RP11-89K21.1 were downregulated in UCEC compared with normal tissues (*P*<0.05). **e-g** miR-24-1-5p (**e**) and miR-503 (**f-g**) correlated with RP11-357H14.17 were downregulated in UCEC compared with normal tissues (*P*<0.05). UCEC: Uterine Corpus Endometrial Carcinoma.

**Table S1 The overall survival of differentially expressed lncRNAs including RP11-89K21.1 and RP11-357H14.17 in UCEC with Kaplan Meier plotter**

| Gene | Ensembl ID | Genome location | *P*-value | Hazard ratio (95% CI） |
| --- | --- | --- | --- | --- |
| RP11-89K21.1 | ENSG00000259439 | chr2:44921077-44939199 | 0.023 | 2.38 (1.1-5.17) |
| RP11-357H14.17 | ENSG00000272763 | chr17:48635923-48647023 | - | - |
| CTD-2314B22.1 | ENSG00000258314 | chr14:19054341-19055551 | 0.074 | 1.7 (0.94-3.06) |
| CTD-2377D24.6 | ENSG00000244649 | chr17:48705203-48707346 | - | - |
| RP11-657O9.1 | ENSG00000240086 | chr3:135373795-135439822 | 0.094 | 1.43 (0.94-2.18) |
| LINC00668 | ENSG00000265933 | chr18:6925478-6928572 | 0.024 | 1.61 (1.06-2.45) |
| AP000892.6 | ENSG00000280143 | chr11:117204967-117210292 | - | - |
| ACTA2-AS1 | ENSG00000180139 | chr10:88932390-88933838 | 0.015 | 1.67 (1.1-2.55) |
| RP11-867G23.10 | ENSG00000254510 | chr11:66409194-66417137 | 0.067 | 1.47 (0.97-2.22) |

**Table S2 Binding miRNAs of RP11-89K21.1 and RP11-357H14.17 with statistical significance predicted with AnnoLnc**

| LncRNA | miRNA | Fold change | *P*-value | FDR |
| --- | --- | --- | --- | --- |
| RP11-89K21.1 | miR-27b-3p | 0.54 | 1.60E-10 | 1.80E-09 |
|  | miR-27b-5p | 0.67 | 1.30E-06 | 9.60E-06 |
|  | miR-4770 | 0.26 | 3.80E-21 | 9.90E-20 |
|  | miR-143-3p | 0.2 | 6.00E-32 | 3.20E-30 |
|  | miR-143-5p | 0.43 | 4.20E-08 | 3.90E-07 |
|  | miR-204-3p | 0.46 | 1.70E-07 | 1.50E-06 |
|  | miR-204-5p | 0.28 | 8.80E-08 | 7.70E-07 |
|  | miR-125a-5p | 0.76 | 0.0082 | 0.031 |
|  | miR-125b-5p | 0.39 | 5.90E-10 | 6.50E-09 |
|  | miR-139-5p | 0.21 | 7.20E-26 | 2.70E-24 |
|  | miR-455-5p | 0.71 | 0.016 | 0.054 |
|  | miR-670-3p | 0.17 | 6.00E-14 | 9.40E-13 |
|  | miR-670-5p | 0.57 | 2.30E-05 | 0.00015 |
| RP11-357H14.17 | miR-27b-3p | 0.54 | 1.60E-10 | 1.80E-09 |
|  | miR-27b-5p | 0.67 | 1.30E-06 | 9.60E-06 |
|  | miR-4770 | 0.26 | 3.80E-21 | 9.90E-20 |
|  | miR-143-3p | 0.2 | 6.00E-32 | 3.20E-30 |
|  | miR-143-5p | 0.43 | 4.20E-08 | 3.90E-07 |
|  | miR-204-3p | 0.46 | 1.70E-07 | 1.50E-06 |
|  | miR-204-5p | 0.28 | 8.80E-08 | 7.70E-07 |
|  | miR-24-1-5p | 0.51 | 4.80E-10 | 5.40E-09 |
|  | miR-503-3p | 0.34 | 0.00018 | 0.00097 |
|  | miR-503-5p | 0.47 | 0.0063 | 0.024 |

**Table S3 Significantly enriched GO annotations (Cellular Components) of RP11-89K21.1 in endometrial carcinoma with Metascape (Top 20)**

| GO | Category | Description | Count | % | Log10(P) | Log10(q) |
| --- | --- | --- | --- | --- | --- | --- |
| GO:0005667 | GO Cellular Components | transcription factor complex | 28 | 7.78 | -12.62 | -9.33 |
| GO:0048471 | GO Cellular Components | perinuclear region of cytoplasm | 37 | 5.09 | -10.89 | -8.20 |
| GO:0070161 | GO Cellular Components | anchoring junction | 30 | 5.31 | -9.33 | -6.74 |
| GO:0005741 | GO Cellular Components | mitochondrial outer membrane | 17 | 9.44 | -9.16 | -6.65 |
| GO:0043235 | GO Cellular Components | receptor complex | 27 | 5.18 | -8.22 | -5.94 |
| GO:0061695 | GO Cellular Components | transferase complex, transferring phosphorus-containing groups | 18 | 7.11 | -7.70 | -5.45 |
| GO:0045177 | GO Cellular Components | apical part of cell | 22 | 5.43 | -7.15 | -4.94 |
| GO:0005942 | GO Cellular Components | phosphatidylinositol 3-kinase complex | 7 | 25.00 | -7.05 | -4.88 |
| GO:0000792 | GO Cellular Components | heterochromatin | 10 | 12.66 | -6.82 | -4.68 |
| GO:0045121 | GO Cellular Components | membrane raft | 19 | 5.81 | -6.70 | -4.60 |
| GO:0031252 | GO Cellular Components | cell leading edge | 21 | 5.02 | -6.29 | -4.29 |
| GO:0035985 | GO Cellular Components | senescence-associated heterochromatin focus | 3 | 75.00 | -4.97 | -3.05 |
| GO:0005635 | GO Cellular Components | nuclear envelope | 20 | 4.20 | -4.86 | -2.96 |
| GO:0017053 | GO Cellular Components | transcriptional repressor complex | 8 | 9.76 | -4.72 | -2.88 |
| GO:0030659 | GO Cellular Components | cytoplasmic vesicle membrane | 26 | 3.32 | -4.36 | -2.59 |
| GO:0098978 | GO Cellular Components | glutamatergic synapse | 16 | 4.48 | -4.33 | -2.57 |
| GO:0097136 | GO Cellular Components | Bcl-2 family protein complex | 3 | 50.00 | -4.28 | -2.54 |
| GO:0033256 | GO Cellular Components | I-kappaB/NF-kappaB complex | 3 | 42.86 | -4.04 | -2.36 |
| GO:0030139 | GO Cellular Components | endocytic vesicle | 14 | 4.67 | -4.04 | -2.36 |
| GO:0098552 | GO Cellular Components | side of membrane | 21 | 3.51 | -3.93 | -2.28 |

**Table S4 Significantly enriched GO annotations (Molecular Functions) of RP11-89K21.1 in endometrial carcinoma with Metascape (Top 20)**

| GO | Category | Description | Count | % | Log10(P) | Log10(q) |
| --- | --- | --- | --- | --- | --- | --- |
| GO:0000987 | GO Molecular Functions | proximal promoter sequence-specific DNA binding | 55 | 8.74 | -27.19 | -23.53 |
| GO:0016773 | GO Molecular Functions | phosphotransferase activity, alcohol group as acceptor | 46 | 6.47 | -17.40 | -14.43 |
| GO:0019900 | GO Molecular Functions | kinase binding | 44 | 5.94 | -15.28 | -12.56 |
| GO:0019838 | GO Molecular Functions | growth factor binding | 21 | 14.89 | -15.15 | -12.48 |
| GO:0019904 | GO Molecular Functions | protein domain specific binding | 38 | 5.34 | -11.79 | -9.20 |
| GO:0003712 | GO Molecular Functions | transcription coregulator activity | 32 | 5.42 | -10.16 | -7.61 |
| GO:0019903 | GO Molecular Functions | protein phosphatase binding | 16 | 11.43 | -9.89 | -7.42 |
| GO:0042803 | GO Molecular Functions | protein homodimerization activity | 32 | 4.95 | -9.17 | -6.74 |
| GO:0005126 | GO Molecular Functions | cytokine receptor binding | 20 | 7.63 | -9.02 | -6.61 |
| GO:0019207 | GO Molecular Functions | kinase regulator activity | 18 | 8.57 | -8.98 | -6.59 |
| GO:0046332 | GO Molecular Functions | SMAD binding | 11 | 14.47 | -8.07 | -5.75 |
| GO:0031625 | GO Molecular Functions | ubiquitin protein ligase binding | 19 | 6.51 | -7.47 | -5.22 |
| GO:0052812 | GO Molecular Functions | phosphatidylinositol-3,4-bisphosphate 5-kinase activity | 4 | 80.00 | -6.74 | -4.52 |
| GO:0050839 | GO Molecular Functions | cell adhesion molecule binding | 24 | 4.76 | -6.69 | -4.48 |
| GO:0047485 | GO Molecular Functions | protein N-terminus binding | 11 | 10.28 | -6.51 | -4.32 |
| GO:0002020 | GO Molecular Functions | protease binding | 12 | 9.16 | -6.49 | -4.32 |
| GO:0016538 | GO Molecular Functions | cyclin-dependent protein serine/threonine kinase regulator activity | 8 | 16.33 | -6.44 | -4.28 |
| GO:0035497 | GO Molecular Functions | cAMP response element binding | 5 | 35.71 | -6.03 | -3.91 |
| GO:0003707 | GO Molecular Functions | steroid hormone receptor activity | 8 | 14.29 | -5.98 | -3.87 |
| GO:0001223 | GO Molecular Functions | transcription coactivator binding | 6 | 22.22 | -5.79 | -3.70 |

**Table S5 Significantly enriched GO annotations (Cellular Components) of RP11-357H14.17 in endometrial carcinoma with Metascape (Top 20)**

| GO | Category | Description | Count | % | Log10(P) | Log10(q) |
| --- | --- | --- | --- | --- | --- | --- |
| GO:0048471 | GO Cellular Components | perinuclear region of cytoplasm | 40 | 5.50 | -14.47 | -11.18 |
| GO:0000307 | GO Cellular Components | cyclin-dependent protein kinase holoenzyme complex | 12 | 28.57 | -13.05 | -10.06 |
| GO:0005912 | GO Cellular Components | adherens junction | 31 | 5.65 | -11.56 | -8.97 |
| GO:0005667 | GO Cellular Components | transcription factor complex | 23 | 6.39 | -9.73 | -7.35 |
| GO:0045121 | GO Cellular Components | membrane raft | 20 | 6.12 | -8.21 | -6.07 |
| GO:0043235 | GO Cellular Components | receptor complex | 24 | 4.61 | -7.34 | -5.28 |
| GO:0031252 | GO Cellular Components | cell leading edge | 19 | 4.55 | -5.83 | -3.82 |
| GO:0031012 | GO Cellular Components | extracellular matrix | 21 | 3.93 | -5.38 | -3.39 |
| GO:0030139 | GO Cellular Components | endocytic vesicle | 15 | 5.00 | -5.21 | -3.25 |
| GO:0005635 | GO Cellular Components | nuclear envelope | 19 | 3.99 | -5.02 | -3.08 |
| GO:0000792 | GO Cellular Components | heterochromatin | 7 | 8.86 | -4.23 | -2.34 |
| GO:0071141 | GO Cellular Components | SMAD protein complex | 3 | 42.86 | -4.19 | -2.33 |
| GO:0098552 | GO Cellular Components | side of membrane | 20 | 3.34 | -4.15 | -2.31 |
| GO:0005741 | GO Cellular Components | mitochondrial outer membrane | 10 | 5.56 | -4.04 | -2.21 |
| GO:0016235 | GO Cellular Components | aggresome | 5 | 12.82 | -3.93 | -2.12 |
| GO:0034399 | GO Cellular Components | nuclear periphery | 8 | 6.15 | -3.64 | -1.87 |
| GO:0005657 | GO Cellular Components | replication fork | 6 | 8.45 | -3.59 | -1.85 |
| GO:0005769 | GO Cellular Components | early endosome | 14 | 3.76 | -3.58 | -1.85 |
| GO:0035631 | GO Cellular Components | CD40 receptor complex | 3 | 27.27 | -3.53 | -1.84 |
| GO:0031256 | GO Cellular Components | leading edge membrane | 9 | 5.17 | -3.46 | -1.77 |

**Table S6 Significantly enriched GO annotations (Molecular Functions) of RP11-357H14.17 in endometrial carcinoma with Metascape (Top 20)**

| GO | Category | Description | Count | % | Log10(P) | Log10(q) |
| --- | --- | --- | --- | --- | --- | --- |
| GO:0004672 | GO Molecular Functions | protein kinase activity | 46 | 7.59 | -22.23 | -18.56 |
| GO:0008134 | GO Molecular Functions | transcription factor binding | 45 | 6.83 | -19.90 | -16.71 |
| GO:0019900 | GO Molecular Functions | kinase binding | 46 | 6.21 | -18.69 | -15.72 |
| GO:0019904 | GO Molecular Functions | protein domain specific binding | 40 | 5.62 | -14.77 | -12.11 |
| GO:0019838 | GO Molecular Functions | growth factor binding | 19 | 13.48 | -13.83 | -11.20 |
| GO:0031625 | GO Molecular Functions | ubiquitin protein ligase binding | 25 | 8.56 | -13.35 | -10.83 |
| GO:0005126 | GO Molecular Functions | cytokine receptor binding | 23 | 8.78 | -12.55 | -10.09 |
| GO:0003712 | GO Molecular Functions | transcription coregulator activity | 29 | 4.92 | -9.41 | -7.09 |
| GO:0002020 | GO Molecular Functions | protease binding | 14 | 10.69 | -8.97 | -6.67 |
| GO:0050839 | GO Molecular Functions | cell adhesion molecule binding | 25 | 4.96 | -8.26 | -6.01 |
| GO:0019902 | GO Molecular Functions | phosphatase binding | 14 | 7.57 | -7.04 | -4.85 |
| GO:0046332 | GO Molecular Functions | SMAD binding | 9 | 11.84 | -6.36 | -4.21 |
| GO:0035497 | GO Molecular Functions | cAMP response element binding | 5 | 35.71 | -6.28 | -4.14 |
| GO:0042803 | GO Molecular Functions | protein homodimerization activity | 25 | 3.87 | -6.19 | -4.07 |
| GO:0008022 | GO Molecular Functions | protein C-terminus binding | 13 | 6.84 | -6.08 | -3.98 |
| GO:0001223 | GO Molecular Functions | transcription coactivator binding | 6 | 22.22 | -6.07 | -3.98 |
| GO:0043425 | GO Molecular Functions | bHLH transcription factor binding | 6 | 21.43 | -5.97 | -3.90 |
| GO:0004712 | GO Molecular Functions | protein serine/threonine/tyrosine kinase activity | 7 | 15.56 | -5.88 | -3.83 |
| GO:0070888 | GO Molecular Functions | E-box binding | 7 | 13.73 | -5.50 | -3.47 |
| GO:0003707 | GO Molecular Functions | steroid hormone receptor activity | 7 | 12.50 | -5.22 | -3.20 |
